# Surgical reconstruction of a W-shaped lower lip after extensive tissue loss and management of human bite wounds: A case report

**DOI:** 10.1016/j.ijscr.2025.111512

**Published:** 2025-06-14

**Authors:** Mongwa Mbikilile Justin, Héritier Baliwa, Bandeke Matabishi, Alumeti Munyali

**Affiliations:** a Surgery Department, Faculty of medicine and public health, Evangelical University in Africa, Bukavu, South Kivu, the Democratic Republic of the Congo; b Surgery Department, Faculty of Medicine, Official University of Bukavu, Bukavu, South Kivu, the Democratic Republic of the Congo

**Keywords:** A human bite, W-plasty, Lower lip reconstruction, Aesthetic, Cosmetic lip surgery, Case report

## Abstract

**Introduction:**

Human bites represent a considerable risk of infection, which negatively impacts the healing and reconstruction of the lower lip. This case report describes the effectiveness of combining surgical W reconstruction of the lower lip with specialized care to manage severe tissue loss due to biting.

**Case presentation:**

A 22-year-old woman was bitten on the lower lip by her neighbor. A clinical examination revealed a wound with significant tissue loss on the right lower lip. To properly reconstruct and suture the wound edges, a W-shaped excision was performed to remove tissue suspected of harboring infection. Additionally, an arched *endo*-buccal counter-incision at the base of the mucosa was made to mobilize the tissue, ensuring lip symmetry. Appropriate treatment was administered for lesions caused by human bites.

**Clinical discussion:**

The surgical W reconstruction of the lip technique is also used for excising tumors of the lower lip. Although there are other techniques for reconstructing the lip following tissue loss, the one described in this case offers an advantage in terms of ease of execution and reduced tissue damage.

**Conclusion:**

After extensive tissue loss in the lower lip of a human being due to a human bite, this case report suggests that W reconstruction of the wound is a therapeutic alternative, giving short-term aesthetic results with a lower risk of infection.

## Highlights

• As human lip bites are frequent, the fusion of aesthetic reconstruction of the lower lip with the codified management of human bite lesions, after the loss of half of its constituent tissues, is necessary to combat superinfection and promote healing.
• The advancement of the W-plasty technique for lower lip reconstruction, combined with an arched counter incision along the lower dental arch, enables mucosal mobilization while preserving tissue integrity, minimizing the risk of creating multiple endobuccal lesions.
• This case report highlights how adapting a surgical technique, originally used for excising certain lower lip tumors, to reconstruct traumatic wounds or human bites with extensive tissue loss alongside key modifications to minimize buccal mucosa damage, can significantly enhance healing and aesthetic outcomes.

## 1 Introduction

The lips play a crucial role in facial aesthetics and functionality. Therefore, repairing them requires addressing both functional and cosmetic imperatives [1,2]. Human bites pose a significant risk of local bacterial infection, with an estimated incidence of 10 to 20 % [3]. Similar to animal bites, healthcare providers must consider the possibility of disease transmission through human bites. The risk of transmitting blood-borne viruses, especially HIV, hepatitis B (which was isolated from carriers' saliva by the presence of hepatitis B DNA [4]), and hepatitis C, should also be discussed by the attending physician, according to some reports [4,5].

The incidence of human bite injuries is underreported as the victims may not wish to expose the circumstances that led to the bite, especially when they occur between spouses and when there could be legal repercussions [6].

African epidemiological data on human bites are imprecise, as there is little documentation and no statistics are kept on a continental scale. Structured interdisciplinary and surgical management of bite wounds is the most important factor in preventing infection [7].

Most studies report on labial surgery, and techniques such as V- or Y-shaped reconstruction, face lift, flap, and other techniques have been described [[8], [9], [10]]. However, these techniques are often used in the context of labial augmentation or loss of substance following resection of a lower lip tumor. Hence, in the context of this case report, the human bite of the lower lip, with all the infectious risks it entails, was at the root of significant tissue loss. These present two major challenges: postoperative infection related to the lesion's etiology and microstomia with lip asymmetry due to an unsuitable surgical technique.

To reconstruct the lower lip following extensive bite tissue loss from the human bite, this case report proposes a surgical approach and techniques for managing human bite wounds to mitigate infection risk and restore lip symmetry.

## 2 Methods

The work has been reported in line with the SCARE criteria [11].

### 2.1 Case presentation

The 22-year-old female patient was transferred to the general referral hospital for reconstruction of the lower lip following a hemorrhagic wound caused by a human bite, with the loss of part of the lip during a brawl. The incident occurred around 7 h before the current consultation. During a fight with her neighbors, the patient was bitten halfway down her lower lip by her neighbor and presented with a bleeding wound and gingivorrhagia, prompting her relatives to transport her to a nearby hospital. Due to a lack of technical resources and a plastic surgeon, they referred the patient to the hospital for cosmetic reconstruction of the lower lip and improved treatment of the human bite.

She has a history of being married for four years, having two children, occasionally drinking, not having diabetes, and having an unclear immunization schedule.

**Fig. 1:**
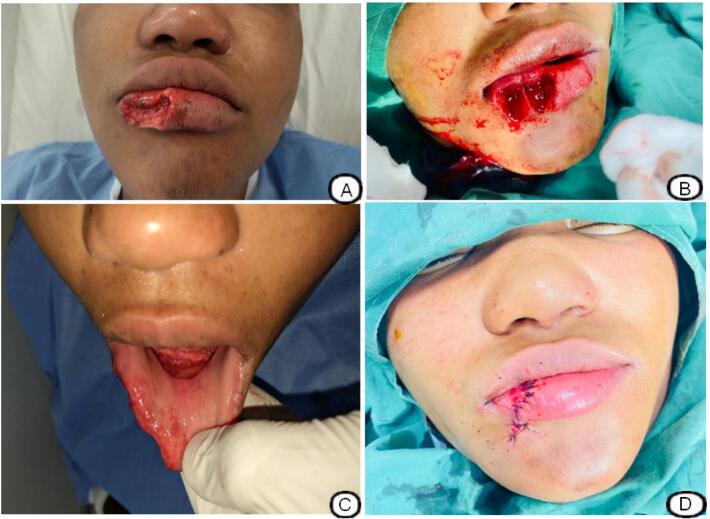
Wound on the lower lip with loss of half of the yeast tissue caused by a human bite, affecting almost half of the lower right lip (vermilion, edge of the lower vermilion, orbicularis muscle, and part of the skin), with irregular edges (A). A W-shaped incision and an excision to regularize the edges of the wound to give it the W shape extended beyond the edge of the lower vermilion (B). The detachment of the lower labial mucosa was slightly accentuated to allow the rest of the lip to be mobilized (C). acting as a counter-incision, the lip was reconstructed by suturing in 2 and 3 planes with 4/0 Vicryl, restoring the almost aesthetic symmetry of the lip and face postoperatively (D).

A physical examination revealed a wound on the lower lip, approximately 3.5 cm long, 2 cm wide, and 1.5 cm deep, with significant tissue loss affecting almost half of the lower right lip (vermilion, edge of lower vermilion, and part of the skin), with irregular edges (Fig. 1A). The patient also expressed her pain and mentioned that she wanted to regain the shape of her lip at all costs. Additionally, vital signs were within normal limits. An endo buccal examination revealed a detachment of the lower lip frenulum and almost all of the mucosa connecting the lower lip to the gingiva. The rest of the physical examination was normal. The diagnosis of a large lower lip wound caused by a human bite with tissue loss was retained.

A surgical treatment was proposed to the patient and accepted; however, she refused any form of additional wound that could be created to perform a graft if necessary. Surgical trimming was performed, with abundant cleansing of the wound with Cetrimide, and surgical reconstruction of the lip was performed under anesthesia after injection of lidocaine in the bloc under ultrasound guidance and through a W-shaped incision and excision (Fig. 1B). This W-shaped incision made it possible to regularize the edges of the wound, which had extended beyond the edge of the inferior vermilion, taking with it part of the skin and inferior orbicularis muscle. The wound was then sutured in 2 and 3 planes with Vicryl 4/0 absorbable suture, the deepest sutured in an inverted U shape, bringing the vermilion border closer, and the detachment of the labial mucosa from the lower cheek was slightly accentuated to allow mobilization of the rest of the lip, acting as a counter-incision (Fig. 1C). The operation was performed by a general surgery resident in his third year of training in minimally invasive techniques.

**Fig. 2:**
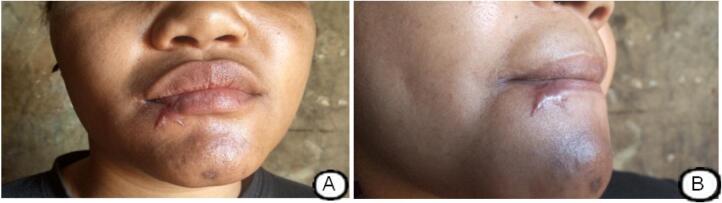
Results after 1 month and 4 days of surgery(A). Presence of a scar at 1 month and 4 days after reconstruction of the lower lip (B).

After the lip was repaired (Fig. 1D), the mucosa was not sewn back together. A sterile pressure dressing was applied, and the use of green betadine for the mouth began. The patient was discharged within 48 h, and at her postoperative follow-up one month and four days after reconstruction, the aesthetic result was satisfactory and the patient declared herself to be satisfied (Fig. 2. A, B).

## 3 Discussion

### 3.1 The principle of surgical management of human bites

The lip, as a functional unit, maintains oral competence, emotive expression, and facilitates speech and feeding [10]. Practically all previous studies suggest the management of human bites follows a protocol, mainly consisting of wound irrigation, debridement [12], and surgical repair [13]. According to previous research, small, thin human bite wounds that cover no more than 25 to 33 % of the lip can be closed in the first instance. Lip reconstruction will then take place when the lesion is greater than this percentage. Further excisions can then be performed [10].

In some cases, V-shaped excision of the entire lip thickness and primary closure of the defect are the most commonly used treatment methods. Alternatively, a pentagon- or W-shaped excision technique can also be used [10]. The management principles for human bite wounds were adhered to in our patient. Regarding surgical technique, there are various methods for reconstructing the lower lip; however, the approach chosen for this case report was based on the evaluation of the wound and the endobuccal characteristics observed in the patient.

### 3.2 W-shape reconstruction surgery, as well as additional surgical options

Despite its widespread use, cosmetic lip surgery can occasionally have disastrous results, particularly when it's intended to alter the lips [14]. Most studies report cases of lip surgery for repair or augmentation [[15], [16], [17]]. However, this study describes the mechanism of lip lesions and tissue loss, which is rarely designated but common in human bite trauma to the lip [18]. A W-plasty of the damaged lower lip and a counter-incision that changes the arc of the mucosa's attachment to the gum line are used in this special treatment to rebuild the lip.

Lip surgery follows basic rules to ensure optimal results. It must respect the mucocutaneous junction, the aesthetic subunits, and the corners of the mouth [1,2,19]. Bernard established the method of using cheek advancement flaps in conjunction with full-thickness wedge excision for lower lip repair in 1853 [20]. Estlander, in 1872, described the transfer of the upper lip to the lower lip at the commissure, while Abbe introduced the central cross-lip flap in 1898 for cleft lips [21,22]. Additional challenges for the maxillofacial surgeon relate to the reconstruction and subsequent repair of lesions from animal and human bites [23]. These different techniques show the complexity of reconstructing the yeast; hence, the need for future researchers to establish a clinical and therapeutic classification to have a protocol that can guide the techniques used.

It has been described how to lift the musculo-mucosal flaps just below the vermilion border [8]. In this case, marking is performed intraorally to create a “W” for each lip treated, with the base of the W oriented toward the labial sulcus and the ends toward the vermilion mucosa junction, and the lateral arms of the W extending to the buccal commissure. The incision is made just below the submucosa, which is used in cases of lip augmentation.

However, in this case report, the advancement flap procedure is not like that of V—Y as described by Jacono and Quatela [8], following changes and the occurrence of tissue loss, with the particularity of combining a W incision to regularize the edges of the traumatized lower lip with an endobuccal counter incision at the gum/mucosa junction, allowing the mucosa to be detached from the gum, thus enabling mobility and repair of the lip.

This W-plasty technique was reported by Chavoin and Garrido-Stowhas [9] in the treatment of tumors and lateral and juxta-commissural loss of substance, described by excising the tumor and approximating the edges in a W-shape, while the internal mucosal plane is simply cut in a V-shape. However, in this study, it was reported that the human bite resulted in a loss of tissue in the lip area, which can also be observed when excising tumors [9], but modifying the technique by creating a mucosal detachment that acts as a counter-incision following the arc of the dental arch, thus enabling the edges to be mobilized and brought closer together without either increasing damage to the mucosa or altering the vascularization and innervation of the lip.

Alternatives for reconstructing the upper or lower lip using regional flaps, taking advantage of the good elasticity of the lips and the presence of folds and margins to camouflage scars, are described in the literature: nasolabial advancement flap, Webster flap, Camille Bernard flap, Gillies fan flap, Karapandzic neurovascular flap, Abbé flap, Estlander flap, heterolabial mucosal flap, transient pedicled tongue flap, and/or *Z*-alignment reconstruction [1,2,9,24], which would explain the usefulness of aesthetic lip reconstruction, as was the case with our patient.

The main advantages of W-shaped reconstruction include its simple execution, minimal need for additional surgery in most cases, and lower risk of microstomia. As outlined in this study, it ensures symmetry, prevents scarring issues, and reduces postoperative complications, even in infection cases, such as human bites. If further surgery is needed, studies indicate that W-plasty remains one of the top reconstruction options [25,26].

The vascularization of the lower lip comprises several arterial, venous, and lymphatic components, including branches mainly of the facial artery, one of which passes through the lower lip, i.e., the inferior coronary artery and the inferior labial arteries [9,15,18,27], which were not damaged in our patient despite the beating and biting of the lip that her husband's patient had suffered after reconstruction of her lower lip; the sensitivity and good vascularization of these lips were intact.

### 3.3 Use of cetrimide

In this study, cetrimide showed satisfactory results, as no signs of infection were noted in the patient. Agents such as povidone‑iodine greater than 1 % have been associated with delayed wound healing and increased infection rates [28]. In a study conducted in England, the authors suggest the use of 0.9 % sodium chloride as the irrigant fluid of choice [13]. However, the amount of cetrimide used for wound cleansing during surgical trimming in this study depended on the lesion and the surgeon.

The human mouth is teeming with bacteria, making human bites a medical emergency, but one that is often underestimated, even though it can lead to serious complications, with infections at the forefront if not treated properly [6]. The use of cetrimide during surgical trimming and betadine green solution for mouthwash would be justified by this, as well as the use of the tetanus toxoid vaccine and antibiotic prophylaxis.

### 3.4 Use of antibiotics for human bite wounds

Bite wounds of the lips are considered complex lesions of the maxillofacial region, usually contaminated with polymicrobial agents that require immediate repair for optimal results [29]. The combination of amoxicillin and clavulanic acid has been reported as the first line of treatment for human bite wounds in the management protocols for England and Wales [13], but we believe that antibiotic use should not be systematic, despite the infectious risk associated with human bite wounds; however, vaccination of the victim and serological testing of salivary secretions and blood from the person who bit the victim can be of vital importance in detecting any possible forms of transmission of viral diseases due to contact with biological secretions.

This case report describes the application of a W-plasty surgical reconstruction technique, incorrectly used as a W-plasty in conjunction with bite-related therapeutic measures, to achieve more or less satisfactory short-term aesthetic results while reducing the risk of infection often seen in bite lesions in humans, which was applied to attenuate the loss of tissue in the lower half of a young woman's lip, restoring satisfactory symmetry to her lower lip as reported by the patient.

### 3.5 Limits and strengths

The strength of this study is the description of the W excision technique used on the patient with two main modifications related to the lesions observed: (i) human bite management measures to limit the risk of infection that could impact proper healing and daily life, while showing that antibiotic therapy or antibiotic prophylaxis for human bites is not systematic and (ii) the performance of a counter-incision at the site of union between the labial mucosa and gingiva to achieve maximum mobilization and restore satisfactory symmetry of the lower lip.

However, the study had focused on a single specific situation, which prevents the conclusions from being generalized, thus constituting a limitation. A randomized trial involving a larger number of patients would improve the results of the treatment mentioned in this study, although we believe it provides sufficient information to improve the management of lip bites.

## 4 Conclusion

Reconstruction of the lower lip bitten by a human being requires the respect of steps due to the infectious risks that can result from these bite lesions. W-plasty and an endobuccal arciform counter incision at the union of the lower lip mucosa and the lower gum can be one of the aesthetic surgical alternatives when aiming to restore lower lip aesthetics after significant tissue loss related to trauma or a bite.

A future meta-analysis comparing various lip reconstruction techniques to establish a protocol classification, guiding technique selection based on observed clinical lesions, would enhance this study.

## Abbreviations

DNA: Deoxyribonucleic Acid

## CRediT authorship contribution statement

Conception: Mbikilile Justin, Alumeti Munyali; Design: Mbikilile Justin and Alumeti Munyali; Litterature search: Mbikilile Justin; Alumeti Munyali; Heritier Baliwa and Bandeke Matabishi; Manuscript preparation: Mbikilile Justin; Manuscript editing: Mbikilile Justin, Alumeti Munyali; Manuscript review: All Authors; Supervision: Alumeti Munyali; Final approval of manuscript: All Authors.

## Patient consent

Written informed consent was obtained from the patient/legal guardian for publication and any accompanying images. A copy of the written consent is available for review by the Editor-in-Chief of this journal on request.

## Ethics approval and consent to participate

Not applicable.

## Guarantor

Mbikilile Justin and Alumeti Munyali are the guarantors of the work and accept full responsibility of the work.

## Provenance and peer review

This article was not commissioned and was peer-reviewed.

## Funding

This work did not receive any specific grant from funding agencies in the public, commercial, or not-for-profit sectors.

## Declaration of competing interest

The authors declare that they have no conflict of interest regarding the publication of this case series.
